# Investigating mitochondrial redox state using NADH and NADPH autofluorescence

**DOI:** 10.1016/j.freeradbiomed.2016.08.010

**Published:** 2016-11

**Authors:** Thomas S. Blacker, Michael R. Duchen

**Affiliations:** a Department of Cell and Developmental Biology, University College London, London WC1E 6BT, UK; b Department of Physics and Astronomy, University College London, London WC1E 6BT, UK

**Keywords:** Nadh, Nadph, Mitochondria, Redox, Fluorescence, Microscopy, FLIM

## Abstract

The redox states of the NAD and NADP pyridine nucleotide pools play critical roles in defining the activity of energy producing pathways, in driving oxidative stress and in maintaining antioxidant defences. Broadly speaking, NAD is primarily engaged in regulating energy-producing catabolic processes, whilst NADP may be involved in both antioxidant defence and free radical generation. Defects in the balance of these pathways are associated with numerous diseases, from diabetes and neurodegenerative disease to heart disease and cancer. As such, a method to assess the abundance and redox state of these separate pools in living tissues would provide invaluable insight into the underlying pathophysiology. Experimentally, the intrinsic fluorescence of the reduced forms of both redox cofactors, NADH and NADPH, has been used for this purpose since the mid-twentieth century. In this review, we outline the modern implementation of these techniques for studying mitochondrial redox state in complex tissue preparations. As the fluorescence spectra of NADH and NADPH are indistinguishable, interpreting the signals resulting from their combined fluorescence, often labelled NAD(P)H, can be complex. We therefore discuss recent studies using fluorescence lifetime imaging microscopy (FLIM) which offer the potential to discriminate between the two separate pools. This technique provides increased metabolic information from cellular autofluorescence in biomedical investigations, offering biochemical insights into the changes in time-resolved NAD(P)H fluorescence signals observed in diseased tissues.

## Highlights

• NAD plays a central role in energy-producing pathways.
• NADP is crucial for maintaining the antioxidant defence.
• The reduced forms, NADH and NADPH, are naturally fluorescent inside living tissues.
• Cellular autofluorescence can be used to investigate NAD(P)H redox state.
• NAD(P)H FLIM allows the contributions from NADH and NADPH to be separated.

## 1 Introduction

The pyridine nucleotide pools, nicotinamide adenine dinucleotide (NAD) and nicotinamide adenine dinucleotide phosphate (NADP), are crucial to the intracellular balance between the generation of reactive oxygen species (ROS) and their neutralisation. The NAD pool participates in processes driving energy homoeostasis, generally associated with the subsequent production of ROS. The NADP pool, meanwhile, plays a primary role in maintaining the antioxidant defences, but in some tissues may also serve as a cofactor in free radical generating reactions [1]. Both NAD and NADP act as “electron carriers”, ferrying reducing equivalents between redox reactions taking place inside the cell. In their oxidised forms, NAD+ and NADP+, both molecules may receive electrons by the addition of a hydride ion, producing the reduced forms, NADH and NADPH. These are intrinsically fluorescent [2], a phenomenon that has been exploited as a label-free method for monitoring the intracellular redox state of living cells and tissues for more than 60 years [3]. The approach represents a potentially powerful tool for interrogating the state of biochemical pathways in cells and tissues, but interpreting changes in autofluorescence in relation to the activity of metabolic pathways remains a challenge [4], [5], [6].

In most cell types, the total pool of NAD (oxidised plus reduced forms) is larger than that of NADP. However, the primary role of NADP is as an electron donor in anabolic pathways. As this requires the pool to be kept in a significantly reduced state, the NADPH/NADP+ ratio is maintained high [7]. In contrast, the role of NAD as an electron acceptor in catabolic pathways requires the pool to be maintained in an oxidised state. Therefore, the NADH/NAD+ ratio is kept low [8]. Thus, while the size of the total NAD pool may be greater than that of the NADP pool, the intracellular concentrations of the reduced forms, NADH and NADPH, are typically of a similar order of magnitude [9]. The spectral properties of these fluorescent forms of the cofactors are indistinguishable [10], [11]. Thus, the mixed signal is often referred to as NAD(P)H [12].

The absorption and emission properties of NAD(P)H were first described by Warburg et al. in the 1930's and 1940's [13]. The construction of instrumentation for the use of NAD(P)H fluorescence as a reporter of redox state in living samples began in the 1950’s with the pioneering work of Chance et al. [14]. In the following years, demonstrations of NAD(P)H fluorescence spectroscopy were performed in isolated mitochondria [15], ex vivo cells [16] and in vivo organs [17]. The development of laser scanning confocal microscopy in the 1970's added spatial resolution to NAD(P)H fluorescence measurements [18], [19], permitting assessment of differences in the redox state between subcellular organelles or different cell types in a complex tissue [20]. In the 1990's, time-resolved fluorescence measurements were applied to NAD(P)H for the first time [21], allowing determination of the lifetime of the autofluorescence in response to changes in redox state. Akin to an optical half-life, the fluorescence lifetime of a molecule is highly sensitive to changes in its local environment [2], suggesting the possibility of quantitative reporting of biochemical changes associated with the NAD and NADP pools from inside living systems without the addition of extrinsic fluorophores. The commercialisation of fluorescence lifetime add-ons to conventional confocal microscopes in the early 2000’s made fluorescence lifetime imaging microscopy (FLIM) widely accessible [22], leading to a rapid growth in the NAD(P)H FLIM literature. The frequent observation of differences in NAD(P)H lifetime characteristics between healthy and neoplastic tissue has now put the development of clinical diagnostic devices at the forefront of this field [23], [24], [25], [26], [27].

Our purpose in this review is to provide both an introduction to the field of NAD(P)H fluorescence imaging and an overview of the relevant biochemical pathways required to interpret the data that such experiments generate. We therefore outline the primary mechanisms of NADH and NADPH production and consumption and introduce the basic photophysics of the cofactors before discussing practical considerations required to apply NAD(P)H fluorescence intensity measurements to probe the mitochondrial redox state. We give an overview of some recent applications of FLIM to NAD(P)H measurements and describe our efforts, using this technique, to separate the contributions of NADH and NADPH to the total autofluorescence signal in several models. As these reduced forms are equally abundant but play contrasting biochemical roles, this method provides crucial insights into the redox balance in living biological systems, beyond those available from measurements of fluorescence intensity alone.

## 2 Biochemical foundations of NAD(P)H fluorescence measurements

### 2.1 The central role of NAD in mitochondrial energy transduction

**Fig. 1:**
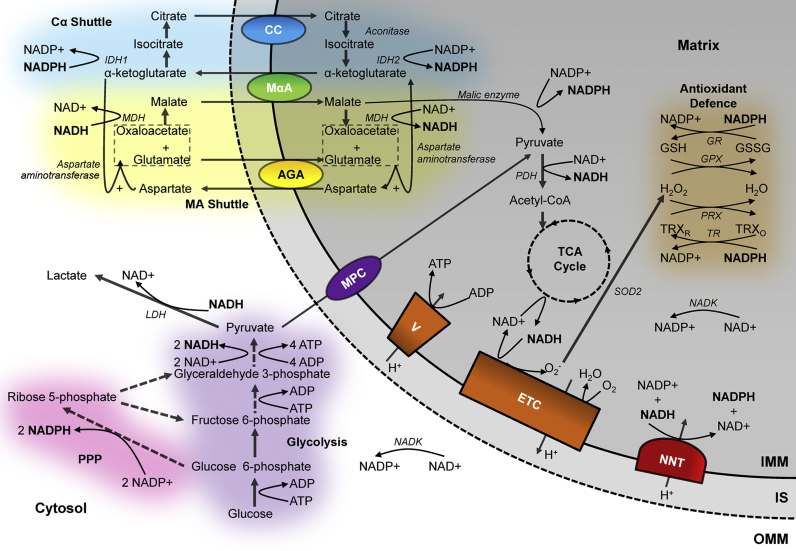
The redox states of the mitochondrial NAD and NADP pools are determined by a range of interconnected pathways. The redox state of cytosolic NAD and NADP, determined by the activities of glycolysis, lactate dehydrogenase (LDH) and the pentose phosphate pathway (PPP), may be indirectly passed to the mitochondria via the malate-aspartate (MA) shuttle and citrate-α-ketoglutarate shuttles (Cα) respectively. These rely on the actions of the aspartate-glutamate (AGA) and malate α-ketoglutarate (MαA) antiporters and the citrate carrier (CC). Inside the mitochondria, pyruvate produced by glycolysis, imported via the mitochondrial pyruvate carrier (MPC), may be converted into acetyl CoA by the pyruvate dehydrogenase (PDH) complex, permitting its oxidation in the TCA cycle to produce NADH. This may then be used to produce ATP at the electron transport chain (ETC) or be converted into NADPH at the nicotinamide nucleotide transhydrogenase (NNT). NADPH may then be used to maintain the glutathione peroxidase (GPX) or peroxiredoxin (PRX) antioxidant systems via the glutathione (GR) and thioredoxin (TR) reductase enzymes.

Glycolysis, taking place in the cytosol, provides the most primitive pathway for converting the chemical potential energy stored in the bonds of glucose into usable energy in the form of adenosine triphosphate (ATP) [28], [29], [30], [31], [32]. As two ATP molecules are used in the early stages of the process, including by the insulin-activated hexokinase to utilise the negative charge of the phosphate group to trap the substrate in the cell [33], the complete conversion of glucose into pyruvate results in a net gain of two ATP molecules [34]. However, as shown in Fig. 1, ATP is not the only molecule to carry energy away from glycolysis. The oxidation of glyceraldehyde 3-phosphate into 1,3-biphosphoglycerate involves passing hydride to NAD+, forming NADH. Glycolysis therefore relies on the availability of NAD+ to ferry electrons away from this reaction. Alongside the activity of phosphofructokinase, the redox state of the cytosolic NAD pool is thus a primary regulator of the glycolytic rate [33], and therefore of mitochondrial substrate supply. To this end, the cytosolic NADH/NAD+ ratio must be maintained low, between 0.01 and 0.05, for glycolysis to proceed [8]. This may be achieved in two ways. Firstly, lactate dehydrogenase may reduce pyruvate into lactate, using NADH to perform the reduction to restore the NAD+ pool [35]. This reaction drives the elevated lactic acid production during anaerobic exercise or hypoxia [36] and is also observed in tumours, even in the presence of sufficient oxygen, in the so-called Warburg effect [34]. Alternatively, the electrons carried by NADH in the cytosol may be transferred to the mitochondria via the malate-aspartate shuttle [37], [38]. While this mechanism is significantly slower at oxidising the cytosolic NAD pool than lactate dehydrogenase, due to the involvement of up to six separate reactions [34], it facilitates the progression of anaerobic glycolysis while providing reducing equivalents to the mitochondria for aerobic metabolism. Indeed, the shuttle plays a significant role in a large number of biological processes, including insulin secretion, cancer cell survival and heart and neurodegenerative diseases [37], [38], [39].

In the mitochondria, oxidative phosphorylation couples ATP production to the energy released by the reduction of oxygen to water using electrons carried by NADH [40], [41], [42], [43]. This produces approximately 28 further ATP molecules from the two molecules of pyruvate derived from the passage of a single glucose molecule through the glycolytic pathway [40]. Pyruvate enters the mitochondria through the pyruvate carrier [44] and is oxidised and decarboxylated into acetyl CoA, producing CO_2_ and NADH [45]. Citrate, formed by joining the acetyl group of acetyl CoA to oxaloacetate, then enters the tricarboxylic acid (TCA) cycle [46]. While this process may classically be viewed as a catabolic prelude to ATP generation by aerobic respiration, the large array of intermediates involved serve as important biosynthetic precursors [34], including for the synthesis of non-essential amino acids and fatty acids [47]. Thus, in proliferating cells, with high biosynthetic requirements, additional carbon sources may be used to increase the flux of the cycle, such as glutamine, which can be converted into both acetyl CoA and TCA cycle intermediates [48], [49], [50]. The full cycle produces one molecule of ATP, two molecules of CO_2_ and, crucially for linking this process to oxidative phosphorylation, three molecules of NADH.

The mitochondria are the site for the universal conversion of all types of fuel substrate into ATP [33], with β oxidation pathways permitting the conversion of fats into acetyl CoA alongside the mechanisms already discussed for the utilisation of sugars and amino acids [51]. NADH links the TCA cycle to cellular energy generation, carrying electrons to the electron transport chain (ETC) on the inner mitochondrial membrane. Upon oxidation to NAD+ by complex I, NADH dehydrogenase, the electrons are passed down the chain to complex IV, cytochrome c oxidase, where four electrons are passed to an oxygen molecule and combined with four protons, producing two molecules of water [42]. The complexes of the ETC couple electron transfer to proton translocation into the intermembrane space, generating the electrochemical gradient across the inner mitochondrial membrane [40], [52] which drives ATP synthesis by the F_1_F_O_-ATP synthase [53], [54]. A single mitochondrial NADH molecule may produce two to five ATP molecules through this mechanism [40], [55], assuming that the protons passed into the intermembrane space cannot leak back into the mitochondrial matrix and that the electrons from NADH are passed along the ETC without loss. In reality, both of these processes may decrease the efficiency of aerobic respiration. Protons may leak across the inner mitochondrial membrane, uncoupling oxidative phosphorylation and generating heat [56], and electrons may also leak from the electron transport chain and pass directly to oxygen, producing superoxide, O_2_^−^, the proximal source of mitochondrial ROS [57]. The deleterious effects of these potentially toxic compounds on nucleic acids, proteins and lipids have been implicated in a range of pathologies from cancer and neurodegeneration to diabetes and atherosclerosis [58]. Electron leak from the respiratory chain is more likely when the complexes are in reduced states [56]. This will occur if NADH is supplied to the electron transport chain at a higher rate than the downstream reduction of oxygen can take place [59], such as following cytochrome c release during apoptosis or under conditions of hypoxia [57]. As mitochondrial hyperpolarisation decreases the rate of respiration [61], an increased membrane potential is associated with increased mitochondrial superoxide production [62]. Uncoupling proteins may decrease the membrane gradient and thus decrease superoxide production [63], and uncoupling has been linked to increased lifespan [64]. However, such an approach may waste energetic resources and limit ATP production, so the cell employs defence systems, maintained by NADPH, to minimise the negative effects of ROS. Thus, while increased NADH levels may increase superoxide production [65], increased NADPH levels may minimise ROS-induced damage. There is therefore a striking contrast between the intracellular roles of these structurally similar cofactors, with their relative abundance relating to the level of oxidative stress within a particular metabolic phenotype.

### 2.2 Maintenance of antioxidant defences by the NADP pool

The neutralisation of superoxide begins with its rapid conversion into hydrogen peroxide H_2_O_2_, by superoxide dismutase (SOD) [66]. Loss of SOD activity causes severe pathology. For example, its knockout in mice causes anaemia, neurodegeneration, severe muscle weakness and reduced lifespan [67]. However, hydrogen peroxide is itself a reactive oxygen species. In contrast to superoxide generated by the ETC, whose negative effects are confined to the mitochondria, hydrogen peroxide carries no net charge, allowing it to diffuse further and inflict damage within the rest of the cell [68], causing DNA strand breaks in the nucleus, leading to mutations [69], and activating DNA repair enzymes which carry negative downstream consequences including the consumption of NAD+ and depletion of mitochondrial substrate supply [70], [71]. Hydrogen peroxide is further neutralised by catalase, which directly decomposes two molecules of hydrogen peroxide into molecular oxygen and two molecules of water. However, the Michaelis constant (K_m_) of catalase for H_2_O_2_ is significantly higher than that of the so-called peroxidases [72]. These thiol-linked antioxidant systems, including glutathione peroxidase (GPX) and peroxiredoxin (PRX), therefore act as the first line of defence against hydrogen peroxide, with catalase playing a role only when the peroxidases are overwhelmed [56]. Two reducing equivalents are required for the reduction of hydrogen peroxide to water in GPX and PRX, provided by glutathione (GSH) and thioredoxin (TRX) respectively. Glutathione reductase and thioredoxin reductase then restore these thiols back to their reduced form to re-establish their antioxidant function. Both enzymes use NADPH as an electron donor and the balance between use of the GPX and PRX systems differs between tissues [73], [74]. Depleted GSH and TRX levels have been associated with a range of pathologies, including cancer, neurodegenerative diseases and alcohol-linked liver disease [75], [76], [77]. Maintenance of the NADP pool in a reduced form is thus crucial to maintaining cell and tissue health [7].

Instead of its oxidation to pyruvate in glycolysis to produce NADH, the oxidation of glucose to ribulose 5-phosphate in the pentose phosphate pathway may alternatively be used to produce cytosolic NADPH [1]. The first of two NADPH-producing enzymes in the pathway, glucose 6-phosphate dehydrogenase, has an affinity for NADP+700-times higher than for NAD+[78], allowing the NADPH/NADP+ ratio in the cytosol to be maintained high (at >3:1), independently of the NAD pool redox ratio [9]. Following this oxidative branch of the pathway [79], continued non-oxidative metabolism of ribulose 5-phosphate may occur, allowing the production of ribose 5-phosphate. The ribose sugar forms part of the structure of ATP, NAD and NADP and, crucially, forms the backbones of RNA and DNA. The pentose phosphate pathway is thus highly active in rapidly dividing cells, such as cancers [80], and the NADPH produced provides the reducing equivalents for lipid synthesis, a crucial requirement for cell proliferation [81]. As is the case for NAD, a shuttle equilibrates the redox state of NADP across the inner mitochondrial membrane, using citrate and α-ketoglutarate [82], [83], [84], as shown in Fig. 1. Mitochondrial NADPH may also be generated by the oxidation of malate to pyruvate by malic enzyme, which plays an important role in insulin secretion [85]. However, the extent to which any NADP-associated dehydrogenase can be used for NADPH production is crucially dependent on the NADP+ concentration. This is generated by the phosphorylation of NAD+, which is uniquely catalysed by NAD kinase [7]. As NADPH levels are determined by the availability of NADP+, NAD kinase plays a vital upstream role in controlling the cellular concentration of NADPH in addition to that played by the individual dehydrogenases [86], [87].

The crucial involvement of NADPH in maintaining the GSH and TRX pools implies a purely antioxidant role for this cofactor, in contrast to NAD and the direct links between its redox state and ROS generation by the ETC. However, in some tissues, NADPH may display pro-oxidative behaviour due to the expression of NADPH oxidase [88]. This multisubunit complex assembles on cell membranes where it catalyses the reduction of molecular oxygen to superoxide, using NADPH as the electron donor. The importance of this enzyme was first recognised in white blood cells [89], which engulf and destroy invading pathogens by phagocytosis. This is thought to be driven by the molar concentration of superoxide produced by the NADPH oxidase at the vacuolar membrane in the respiratory burst [90]. A whole family of NADPH oxidases have been identified and appear to play physiological signalling roles [91]. ROS signalling mediated by the NADPH oxidase is involved in wound healing, regulation of renal blood flow and the generation of otoconia in the ear, to name but three of many [89]. The balance between the pro- and anti-oxidant roles of NADPH is thus dependent on the relative expression of NADPH oxidase and the peroxidases. NADPH oxidase is able to produce superoxide at a maximal rate around five times greater than a single glutathione reductase enzyme can utilise NADPH for antioxidant purposes [92], [93], [94]. However, the K_m_ for NADPH in glutathione reductase is much smaller than that of NADPH oxidase (16 µM vs. 110 µM [94], [95]). At physiological NADPH concentrations (~100 µM [96]), NADPH will only power pro-oxidative reactions to a greater extent than anti-oxidative reactions when the concentration of NADPH oxidase is more than approximately a third of that of the peroxidases, based on Michaelis-Menten kinetics. A search of proteomics data [97] reveals that such levels are only encountered in phagocytic white blood cells such as neutrophils and monocytes, where the balances of NADPH oxidases to peroxidases are around 100 and 1 respectively [98], [99]. In all other tissues, including brain, liver, kidney, heart and lung, peroxidases outnumber members of the NADPH oxidase family (NOX1–5 and DUOX1–2) by around 10 to one [98], [100]. Thus, in the majority of tissues, NADPH primarily acts to prevent, rather than promote, the effects of ROS.

### 2.3 Interconnectivity of NAD and NADP pools

While dehydrogenases associated with the TCA cycle or the citrate-α-ketoglutarate shuttle may contribute, the primary pathway for the maintenance of a reduced NADP pool inside the mitochondria in a large number of cell types involves the direct passage of reducing equivalents from NADH to NADP+ by the mitochondrial nicotinamide nucleotide transhydrogenase (NNT) [101]. Like ATP synthase, the forward reaction of this inner-membrane protein is powered by the translocation of protons into the mitochondrial matrix [102]. A loss-of-function mutation of the NNT in C57BL/6J mice, first recognised in 2005 [9], has helped to establish the importance of this enzyme [103]. NNT expression differs between cell types, being highest in the heart and kidney [104]. Approximately half of the mitochondrial NADPH in the brain is believed to depend on the action of the NNT, and its inhibition causes significant oxidative stress [73]. Thus, this enzyme allows the reducing power produced by the citric acid cycle to be split into an ATP-generating portion as NADH, and an antioxidant portion, used to neutralise the ROS generated during the routine action of the electron transport chain, as NADPH [103], [105]. Alongside this direct link inside the mitochondria, the NAD and NADP pools are also linked in the cytosol by interactions between the pentose phosphate pathway and glycolysis. The non-oxidative branch of the pentose phosphate pathway supports conversion of the ribulose 5-phosphate produced by the oxidative branch into various glycolytic intermediates by a network of enzymes [80], [106]. This provides pathways that control the relative production of NADH, NADPH, ATP and ribose 5-phosphate depending on the instantaneous metabolic requirements of the cell [79], [107], and regulation occurs through feedback between metabolome and transcriptome [108]. The precise pathways utilised will cause varying effects on the redox states of both the NAD and NADP pools.

The key role of the NNT and the network of interactions taking place in cytosolic glucose metabolism highlight that the pathways involved in maintenance of the NAD and NADP pools in their separate redox states are highly interconnected. Indeed, in addition to the malate-asparate and citrate-α-ketoglutarate shuttles providing separate transmission of NAD and NADP redox state between cytosol and mitochondria, a pyruvate-malate shuttle in which the redox state of the cytosolic NADP pool is coupled to that of the mitochondrial NAD pool has also been observed [109]. Additional complexity in these redox networks also arises from the reversibility of a number of the reactions. For example, during ischaemia, the citric acid cycle may reverse and consume NADH [39], the NNT may oxidise NADPH to produce NADH when the membrane potential is collapsed [104] or lactate dehydrogenase may reverse, using lactate as a metabolic substrate, producing NADH in the cytosol alongside pyruvate for aerobic ATP production [110]. Indeed, it has been suggested that lactate secreted by astrocytes may serve as the primary energy source for neurons in the brain [111]. Thus, the highly contrasting intracellular roles of the NAD and NADP pools and their separate redox states are supported by a complex and interconnected network of pathways.

### 2.4 Intrinsic fluorescence of reduced NADH and NADPH

The light absorption properties of NADH and NADPH are identical; spectra peak at 340 nm with a full width at half maximum (FWHM) of ~60 nm [10]. The molecular orbitals involved in this excitation are localised to the nicotinamide ring of the molecule, the redox-active region [112]. Quantum-chemical calculations have shown that removal of the hydride ion carried by NADH or NADPH, forming NAD+ or NADP+, causes the energy of the highest occupied molecular orbital (HOMO) to decrease by 2.96 eV. However, the energy of the lowest unoccupied molecular orbital (LUMO) decreases by only 1.99 eV, meaning the energy difference between the two states involved in the light-induced transition is 0.97 eV larger than that of the electron-carrier in its reduced form. This is caused by the increased positive charge of the carbon atoms bonded to the nitrogen in the nicotinamide ring in NAD(P)+ compared to NAD(P)H [11]. As a result, HOMO to LUMO excitation requires more energy in the oxidised cofactors and their absorption spectrum is blue-shifted relative to their reduced counterparts, peaking at around 220 nm. Optical investigation of NAD+ and NADP+ is not practical inside living tissues as absorption by and subsequent mutation of DNA will be significant at these ultraviolet wavelengths [114]. However, NADH and NADPH represent viable targets as their absorption lies in a window between that of DNA to the blue and flavin, a similarly abundant fluorescent redox cofactor, to the red [115].

**Fig. 2:**
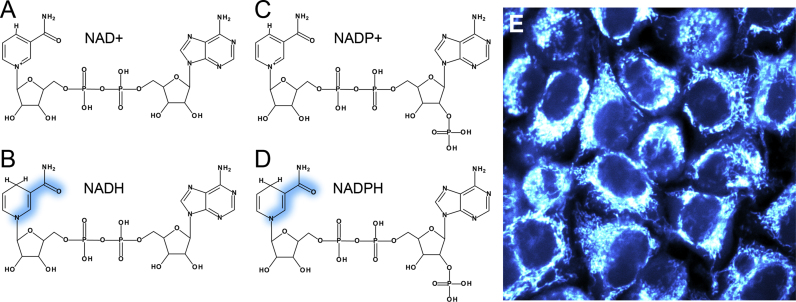
NAD+ and NADP+(A and C) differ from NADH and NADPH (B and D) by the addition of a hydride ion to the nicotinamide ring of the molecule. Absorption of light by NADH and NADPH causes a shift in electron density from the nicotinamide nitrogen towards the oxygen of the amide group (shown in blue). As this group is identical in the two cofactors and lies far from the adenine end of the molecule where the phosphate group exists in NADP not NAD, the spectral characteristics of the two molecules are the same. The combined fluorescence observed in live samples, such as HeLa cells (E), is often labelled NAD(P)H.

Due to their ~10^4^ times larger mass, the nuclei of the molecules remain static over the femtosecond timescales at which electronic excitation takes place [116]. As excitation of NADH and NADPH shifts electron density from the nitrogen of the nicotinamide ring towards the amide group [112], shown in Fig. 2, bond strengths become altered. The atoms will readjust to their lowest excited state potential energy configuration on the picosecond timescale, with energy liberated to the surrounding medium [117]. Fluorescence occurs when the excited molecule relaxes back to the ground state by emitting a photon, with the vibrational energy lost to the surroundings in the excited state causing the light emitted to be of longer wavelength than the light absorbed. The emission spectrum is therefore red-shifted relative to the absorption spectrum [118]. As with the absorption spectra, the emission spectra of NADH and NADPH are identical, peaking at 460 nm with FWHM 100 nm [10]. The shared absorption and emission properties of the two electron-carriers give rise to the combined signal being labelled as NAD(P)H.

Fluorescence is only one of a number of possible routes by which a molecule in the excited state can return to the ground state [119]. Alternatively, the energy of excitation can be transferred during collisions with surrounding molecules or other molecular groups attached to the fluorophore in a process known as quenching [120]. Furthermore, the energy absorbed may instead initiate motion within part of the molecule to return it to its ground state configuration. This process is known as internal conversion [2]. As quenching and internal conversion do not involve the emission of a photon, both are examples of non-radiative de-excitation processes. In the absence of non-radiative de-excitation, a particular molecular species will remain in its excited state for an amount of time dictated by the intrinsic electronic structure of the molecule [121]. The introduction of non-radiative decay pathways will increase the rate at which the molecules return to the ground state, decreasing the average dwell time in the excited state. This interval, typically of the order of nanoseconds, is known as the fluorescence lifetime [122]. As the rates at which quenching and internal conversion occur will reflect the immediate surroundings of the molecule, changes in fluorescence lifetime can be used to infer changes in the environment with which a fluorescent species is interacting [2], [120], [123], [124], [125].

Aqueous NADH solutions at room temperature display two fluorescence lifetimes of approximately 0.3 ns and 0.7 ns [126]. These values are identical to the fluorescence lifetimes of solutions of NADPH [2], which is unsurprising given the distance of the phosphate group from the chromophoric nicotinamide ring. These apparently homogeneous samples must therefore consist of two distinct fluorescent species. Unresolved disagreement exists in the literature as to whether these two species correspond to contrasting interactions between the nicotinamide and adenine rings in extended and folded configurations of the cofactor [127], [128] or reflect competing excited state processes taking place on the nicotinamide ring alone [112], [129]. However, it has long been established that binding to enzymes increases the fluorescence lifetimes of NADH and NADPH to values between 1 ns to 6.5 ns, with the precise lifetime increase dictated by the enzyme to which it binds [127], [130], [131], [132], [133], [13], [3], [96]. The cause of the lifetime increase has previously been assumed to be due to the cofactor being shielded from quenching in the binding site [135]. However, this would imply that an unrealistically large (molar) concentration of quenchers were present in the surrounding medium [136] and so interactions between the cofactor and the binding site must also contribute [137]. Restricting the conformational freedom of the nicotinamide moiety has been shown to dramatically increase the lifetime of NAD(P)H by shutting down internal conversion pathways [2], [138] and so differences in lifetime when bound to different enzymes are likely mediated by the rigidity of each enzyme-cofactor complex [139].

The ratio of photons emitted as fluorescence to total photons absorbed is known as the fluorescence quantum yield of a molecule. NADH and NADPH each possess a relatively low quantum yield of 2% when isolated in solution [127]. Enzyme binding decreases the likelihood of return of an excited molecule to the ground state via a non-radiative pathway and leaves the radiative rate largely unaffected [133]. This increases the quantum yield in proportion to the increase in lifetime. Tailor-made fluorescent dyes will be designed and synthesised to possess high quantum yields in order to maximise their brightness. For example, the dyes rhodamine 6G and fluorescein have quantum yields of 95% and 97% respectively [140]. Achieving such high quantum yields for NAD(P)H would require an enzyme to increase the lifetime of the cofactor to around 20 ns, significantly longer than has ever been observed. Increases in lifetime to between 1 ns and 6.5 ns correspond to increases in quantum yield to only 5% and 33% respectively. Nevertheless, with laser excitation and sensitive detectors, this metabolic cofactor still represents a viable target for studying mitochondrial redox state using fluorescence imaging.

## 3 Practical application of live-cell NAD(P)H fluorescence

### 3.1 NAD(P)H fluorescence in live cells and tissues

While the historical development of NAD(P)H autofluorescence monitoring was carried out on custom-built fluorometry systems [13], [14], the technique has been made more accessible by the wide availability of confocal microscopy in the 21st century laboratory. High quality objective lenses and precise laser control by galvanometer mirrors ensures that the fundamental restriction to the lateral resolution of the obtained images is the size of the diffraction-limited focused illumination spot that is scanned across the sample [141]. For visible light, this corresponds to around 250 nm. As the width of a mitochondrion lies between 500 and 1000 nm [142], mitochondrial morphology can be successfully resolved with confocal microscopy [143]. In the case of NAD(P)H fluorescence, this allows the response of the mitochondrial redox state to an external perturbation to be differentiated from that of the cytosol [20]. Spectrally-distinct mitochondrially-targeted dyes, such as tetramethylrhodamine methyl ester (TMRM) may be used to aid the identification of organelle-specific regions of interest [4]. The “confocal pinhole”, an aperture placed in front of the detector that rejects out of focus light and gives confocal microscopy its name, allows images to be taken at different depths through the sample [144]. Following this “optical sectioning”, the slices can be reconstructed into a three-dimensional image, permitting the full spatial organisation of the mitochondrial network inside a live cell or tissue to be characterised [145].

Performing fluorescence imaging of NAD(P)H may require the addition of an ultraviolet laser to an existing confocal system, due to the blue-shifted absorption wavelength of the cofactor in comparison to most visible fluorophores. For example, our setup consists of an inverted LSM 510 laser scanning confocal microscope (Carl Zeiss) with 351 nm excitation provided by an argon ion laser (Coherent Enterprise UV), while the emitted signal is measured after passing through a 435–485 nm bandpass emission filter to exclude the small amount of contaminating flavoprotein fluorescence that may occur at this wavelength [146]. As the transmittance of microscope optics decreases rapidly at wavelengths below 400 nm, it is necessary to use quartz optics in the excitation path. Additionally, the relatively weak fluorescence of NAD(P)H compared to conventional fluorophores may require sacrifice of axial resolution for signal intensity by using the largest confocal pinhole available. Two-photon excitation offers an alternative to the application of a UV laser [147]. Here, two photons are absorbed effectively simultaneously to provide the total energy required for transition from the ground to excited state [148]. Each photon provides half the energy needed for the transition, so the wavelength of the excitation light required doubles to 700–740 nm, negating the need for quartz optics while maintaining the emission at 460(±50) nm. However, as two-photon absorption is a low probability event, significant excitation will only take place within the focal volume of the objective lens, with the high photon flux output by a pulsed, typically Ti: sapphire, laser also required [149]. This offers the advantage of inherent optical sectioning without the need for a pinhole, with axial resolutions of around 500 nm comparable with those achievable using single photon confocal microscopy [147], [150]. However, this may be a drawback for autofluorescence imaging where acquisition times may need to be increased to achieve the same signal levels as for single-photon excitation, lowering the temporal resolution of the measurement. Two-photon excitation also allows images to be acquired at a greater depth into a thick sample compared to single-photon methods (up to 1 mm, compared to 100 µm [149]) allowing mitochondrial redox state to be investigated in complex living tissue preparations as well as simple cell culture models.

At each pixel of an image, the level of NAD(P)H fluorescence will reflect the concentration of the reduced forms of the two cofactors at that location. This is governed by the total size of the NAD and NADP pools, and the balance of oxidation and reduction reactions. Assuming that both NAD biosynthesis and reduction of NAD+ to NADH by the TCA cycle remain constant over the timescale of the changes (within seconds [115]), the observed signal will reflect changes in the NAD(P)H/NAD(P)+ ratio. This can be clearly demonstrated by considering the effect of alterations in the rate of the ETC on the redox state of the mitochondrial NAD pool. Indeed, such correlations were used by Chance et al. to first identify NAD(P)H as the source of live-tissue autofluorescence in this spectral region [17]. Decreased ETC activity, for example following a decreased oxygen supply, will limit the rate of oxidation of NADH at complex I, increase the NADH/NAD+ redox ratio, and increase mitochondrial NAD(P)H fluorescence. Conversely, increasing ETC activity, such as by uncoupling, will increase oxidation of NADH at complex I, decrease the mitochondrial NADH/NAD+ ratio and decrease NAD(P)H fluorescence [115]. The activities of redox-associated pathways can therefore be qualitatively assessed by measuring the change in the NAD(P)H fluorescence level in response to a carefully chosen external perturbation. For example, taking advantage of the spatial resolution afforded by modern confocal imaging, the activity of the malate-aspartate shuttle could be estimated by observing the change in NAD(P)H fluorescence distribution upon the application of its inhibitor aminooxyacetic acid (AOAA) [38]. If the shuttle was highly active, its inhibition would decrease the fluorescence of the mitochondria coupled with an increase in the rest of the cell. Other examples of monitoring the change in NAD(P)H intensity in response to an environmental change include the demonstration that β-amyloid causes astrocytic oxidative stress in Alzheimer’s disease [70], spatial variations in the redox response to hypoxia in cardiomyocytes [151] and hyperactivation of the NAD-consuming DNA repair enzyme poly-adenosine ribosyl polymerase (PARP) during glutamate excitotoxicity [71].

In systems in which it is necessary to compare two separate biological preparations, direct comparison of the NAD(P)H fluorescence intensity will not provide a comparison of their individual redox states, as the total NAD(P) pool may differ between the two samples. For example, two cell types with identical resting NAD(P)H levels may have drastically different NAD(P)+ levels. As the mitochondrial NADH/NAD+ redox ratio, and not the absolute NADH level, will reflect the balance between mitochondrial NADH supply and demand, linked to the level of superoxide production at the respiratory chain [57], a protocol for measuring this quantity in living cells has found widespread use. By applying drug treatments chosen to maximally reduce or maximally oxidise mitochondrial NAD, direct comparisons of redox state can be made between separate samples. Application of the uncoupler carbonyl cyanide 4-(trifluoromethoxy)phenylhydrazone (FCCP) will cause maximal oxidation of the mitochondrial NADH pool by the action of NADH dehydrogenase, whereas inhibition of complex IV of the electron transport chain using cyanide will stop respiration, maximally reducing the mitochondrial NADH pool. The intensity of NAD(P)H fluorescence observed in response to FCCP and cyanide treatment then defines a dynamic range for the comparison of the emission intensity under the initial untreated conditions. This resting intensity can be normalised and scaled between the minimum and maximum values obtained with the drug treatments to give an approximate value of the mitochondrial NADH/NAD+ ratio [115]. This approach has been applied in a wide range of contexts, ranging from the role of PINK1 in neurodegeneration in Parkinson's disease [152] and the effects of IF1 protein and polyphosphate expression on mitochondrial function [153], [154], to differences in mitochondrial redox state between kidney tubules [155] and role of the maternal diet in oocyte metabolism [156].

In principle, if compounds could be applied that unequivocally caused maximal oxidation and reduction of the separate populations of cytosolic NAD, cytosolic NADP and mitochondrial NADP, the redox ratios of these cofactor pools could be estimated in a similar manner to the FCCP/cyanide assay for mitochondrial NAD. However, the established links between the pathways that maintain each of these four populations in their respective redox states mean that any external perturbation is likely to influence pools other than those targeted. For example, while FCCP causes complete collapse of the mitochondrial membrane potential, computational modelling [157] suggests that decreases of just 25 mV, from a starting value of around 180 mV [158], are sufficient to cause NNT reversal [104]. The model predicts that around 50% of the total NADPH would be oxidised by this reversal, in addition to a near-total oxidation of the NAD pool. This additional NADPH oxidation upon FCCP application would cause the observed baseline NAD(P)H signal to be lower than if NADH alone was being oxidised, causing an overestimation of the NADH to NAD+ ratio. Based on the pool sizes and redox ratios quantified by Ronchi et al. [9], the true ratio could be up to 46% smaller than that implied by the measurements. Efforts have been made to isolate the contribution of NADP to the total NAD(P)H signal by applying hydrogen peroxide to oxidise NADPH to NADP+ through the action of glutathione reductase and thioredoxin reductase [104]. However, as NADPH represents a conserved species with NAD kinase being the key regulator of its concentration [87], the array of pathways for its production will be upregulated under oxidative stress, making it difficult to fully oxidise the NADP pool [160]. For decades a method has been required for quantitative measurement of the relative contributions of NADH and NADPH to the total NAD(P)H signal to avoid these complications associated with the off-target effects of external perturbations. As seen here, inhibitor-based methods for assessing the relative contribution of the two pools provide only qualitative comparisons, in an era where quantitative biochemical assays are becoming ever more crucial [161]. The application of fluorescence lifetime imaging microscopy to the study of NAD(P)H seems at last to provide a potential solution to these drawbacks [4].

### 3.2 Differences in fluorescence lifetime of NADH and NADPH in live cells

**Fig. 3:**
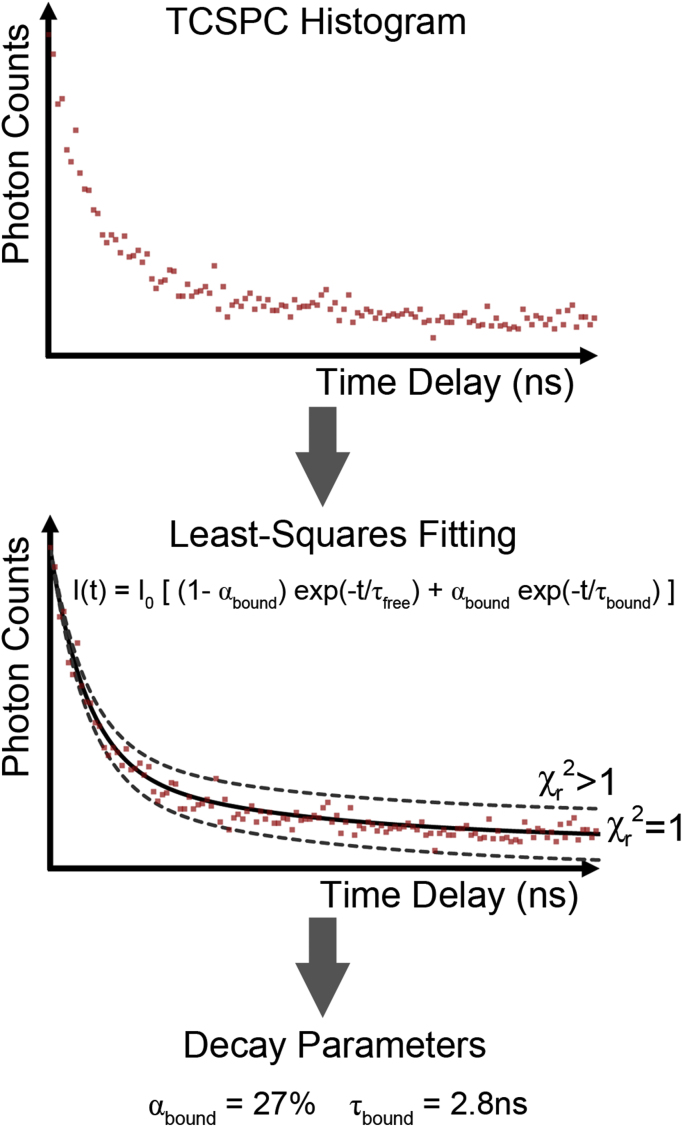
Fluorescence lifetime imaging microscopy (FLIM) records a time-correlated single photon counting (TCSPC) histogram at each pixel of the image. Fitting of a biexponential decay model to this data, using the minimisation of the reduced chi-squared ($\chi_{R}^{2}$) statistic as a goodness-of-fit parameter, gives the average lifetime of bound NAD(P)H species $\tau_{\mathrm{bound}}$ at that pixel, alongside the fraction of the NAD(P)H that is bound to enzymes $\alpha_{\mathrm{bound}}$.

Lifetime measurements had been employed as a sensitive probe of the excited state behaviour of solutions of fluorescent molecules since the mid 20th century [162]. However, these approaches were first adapted to perform measurements across two spatial dimensions in the 1990’s, with the acronym FLIM introduced to describe an apparatus used to acquire “images in which the contrast is based upon the fluorescence lifetimes rather than upon local probe concentrations or local intensity” [163]. In 1992, the fluorescence lifetime of NAD(P)H within live biological samples was first measured [21]. This was performed on suspensions of yeast in a cuvette and employed the time-correlated single photon counting (TCSPC) technique [22], shown schematically in Fig. 3. Now the standard approach in NAD(P)H FLIM, this method involves repeatedly exciting the sample with a pulsed light source. The illumination intensity is reduced so that, on average, only one in 100 pulses causes the emission of a single fluorescence photon, which is registered by a sensitive detector. The time delay between the incoming pulse and the emission of fluorescence is recorded and the corresponding bin on a histogram of delay times is incremented [125]. When the arrival times of sufficient numbers of photons (typically more than 2000) have been registered, the histogram will provide sufficient data to allow measurement of the exponential rates of decay of the excited state population. This is achieved by fitting decay models to the data, providing the time constant of the exponential decay corresponding to the fluorescence lifetime of the sample [164]. In the case of NAD(P)H in the live yeast suspensions, a single lifetime did not adequately describe the observed fluorescence decay [21], as judged by the value of the reduced chi-squared statistic. For a perfect agreement between model and data, this statistic is equal to one [2]. In light of the NAD(P)H photophysics described above, the observation of more than one lifetime inside living biological samples is not surprising, indicating the presence of both freely diffusing and enzyme bound NAD(P)H species.

FLIM performs the TCSPC method across a confocal image, allowing the lifetimes of the fluorescent species present to be determined at each pixel. While NAD(P)H displays two lifetimes when free in solution and a wide range of different lifetimes when bound to different enzymes [127], [130], [131], [132], [133], [13], [3], [96], NAD(P)H FLIM studies typically resolve two lifetimes at each pixel, of approximately 0.4 ns and 2–4 ns [4], [96], [165], [166]. This results from signal-to-noise constraints imposed by the requirement to maintain the integrity of the live biological sample being imaged, necessitating very low laser intensities and short imaging times. The values themselves correspond to a concentration-weighted average of the 0.3 ns and 0.8 ns free lifetimes, labelled $\tau_{\mathrm{free}}$, and an average of the specific enzyme bound species present at that location, labelled $\tau_{\mathrm{bound}}$ [4]. As fluorescence lifetime measurements correlate with changes in the local environment of the fluorophore, intracellular NAD(P)H lifetimes were quickly recognised to be sensitive to changes in the metabolic state of a tissue. For example, in 1996 it was observed that the fraction of NAD(P)H fluorescence emitted by enzyme-bound species, labelled $\alpha_{\mathrm{bound}}$, would decrease as the availability of oxygen was decreased [167]. In a model of oral carcinogenesis, Skala et al. showed that the value of $\tau_{\mathrm{bound}}$ itself was significantly shorter in neoplastic tissue than in adjacent healthy tissue, decreasing from 2.03 ns in control regions to 1.6 ns in precancerous regions, indicating a difference in the distribution of enzymes that the NAD(P)H present was binding to between the two tissue types [165]. The authors then demonstrated that mimicking hypoxia by application of cobalt chloride to cell cultures [168] also caused a significant decrease in the enzyme bound lifetime, perhaps indicating a Warburg-like inhibition of oxidative phosphorylation upon carcinogenesis. Alongside recent work correlating NAD(P)H fluorescence decay characteristics to phosphorescence-based measurements of oxygen concentrations [169], such observations demonstrate clear potential for monitoring metabolic state using NAD(P)H FLIM.

Between the early demonstrations in the 1990's and the present day, more than 1500 NAD(P)H fluorescence lifetime imaging studies have been published (Google Scholar), describing changes in the lifetime characteristics of live-cell NAD(P)H fluorescence in situations ranging from the onset of apoptosis [166], [170], [171], necrotic deterioration of skin [172] and wound healing [173] to stem cell differentiation [174], blood-glucose sensing [175] and aggregation of α-*syn*uclein in Parkinson’s disease [176]. However, until recently, the mechanisms linking the known metabolic shifts in these biological models to the changes in NAD(P)H lifetime were largely unknown, limiting their value as a biomedical assay. Combining the Warburg hypothesis of 1924 with the frequent observation of differences in NAD(P)H lifetime between healthy and cancerous tissue leads to the popular assumption that NAD(P)H lifetime changes reflect changes in the balance of ATP production between oxidative phosphorylation and glycolysis [177]. However, oncogenic metabolic transformations are now known to be significantly more complex than a simple shift from aerobic to anaerobic ATP production, involving changes in both NAD- and NADP-associated pathways [81]. Indeed, recent studies have suggested that the relative rates of glycolysis and oxidative phosphorylation do not directly dictate the intracellular NAD(P)H fluorescence lifetime and that any relationship between these metabolic pathways and the fluorescence decay characteristics of NAD(P)H in the tissue may be considerably more nuanced [4], [178].

Despite its relatively similar abundance [9], [87], identical spectral characteristics [10], equally important role in intracellular metabolism [1] yet independent enzyme binding sites [179], the involvement of NADPH alongside NADH in determining the intracellular NAD(P)H fluorescence lifetime has largely been ignored. However, in 2008, Niesner et al. performed a novel NAD(P)H FLIM study on granulocytes exposed to Aspergillus fumigatus [180]. As discussed above, white blood cells contain sufficient NADPH oxidase for NADPH to act as a pro-oxidant [97], [98], [99]. Contact between the pathogen and the cell membrane initiates assembly of the NADPH oxidase and locally increased glucose flux through the pentose phosphate pathway, allowing production of the molar concentrations of superoxide required to break down the fungus [90], [181]. The authors observed the lifetime of enzyme bound NAD(P)H to be approximately 2 ns within the majority of the granulocytes, but localised regions of the cytosol in contact with the fungus displayed increased enzyme bound lifetimes of 3.7 ns. This offered the possibility that increased NADPH levels are associated with increased enzyme bound NAD(P)H fluorescence lifetimes.

To systematically investigate the role of NADPH in determining the time-resolved fluorescence of intracellular NAD(P)H, we recently compared the NAD(P)H fluorescence lifetime in cell lines in which NAD kinase was either knocked down or overexpressed [4], [87]. Knockdown caused a 3-fold decrease in NADPH concentration relative to control cells and was associated with an enzyme-bound NAD(P)H lifetime of 2.7 ns, whereas overexpression caused a 4- to 5-fold increase in NADPH relative to control and an increase in the lifetime of enzyme bound NAD(P)H to 3.8 ns. Selectively unbinding NADPH from its enzymes by exposure to the competitive inhibitor epigallocatechin gallate (EGCG) decreased the lifetime in the overexpressing cells back to 3.1 ns [182]. As altered NAD kinase expression caused little effect on NADH levels [87], these data implied that intracellular bound NADPH shows a significantly longer fluorescence lifetime than bound NADH. These results therefore suggested that the lifetime of enzyme-bound NAD(P)H in a particular cell type is determined by the balance of NADPH to NADH. In support of this hypothesis, we observed significantly higher glutathione levels in cell types of the mammalian cochlea in which the enzyme bound NAD(P)H lifetime, and therefore the NADPH/NADH ratio, was increased [4], [183]. As the relative sizes of the NADPH and NADH populations dictates the balance between ROS defence and production, an increased enzyme-associated NAD(P)H fluorescence lifetime may therefore reflect an increased antioxidative capacity and subsequently decreased oxidative stress.

To further test the hypothesis that the NADPH/NADH balance determines the enzyme-bound intracellular NAD(P)H lifetime, we compared the results of FLIM measurements on cardiomyocytes from C57BL mice in which the NNT was present or absent [104]. While the enzyme-bound NAD(P)H lifetime was 2.5 ns in both cell types, addition of FCCP significantly increased this value to 2.8 ns in the cells lacking the NNT only. The lack of change in the NNT-expressing cells was interpreted as a reflection of the equal oxidation of NAD and NADP due to the simultaneous action of complex I of the respiratory chain and the reverse action of the NNT, oxidising NADPH to produce NADH in response to the FCCP-induced collapse of the mitochondrial membrane potential. In the absence of the NNT, NADH was still oxidised in response to FCCP, but there was no simultaneous oxidation of NADPH, causing the lifetime to increase. Inhibition of the electron transport chain in these cells with cyanide significantly decreased the bound NAD(P)H lifetime, to 1.9 ns in the absence of NNT and 2.1 ns in its presence. As inhibition of respiration causes accumulation of NADH, these results also suggest that the NADPH/NADH balance defines the enzyme bound NAD(P)H lifetime. The slightly larger value with the NNT present likely reflects its primary action in converting part of the abundance of NADH into NADPH. These results helped to demonstrate that reversal of the NNT increases oxidative stress during pathological cardiac overload, as mice lacking this enzyme were protected from heart failure [104]. Such findings could lead to novel strategies for targeting diseases mediated by mitochondrial ROS production, highlighting the utility of NADH and NADPH fluorescence as an intrinsic biochemical probe.

### 3.3 Separating NADH and NADPH using fluorescence lifetime imaging

The results discussed above imply that a rigourous understanding of the differential intracellular photophysics of NADH and NADPH will permit the quantitative assessment of their concentrations using NAD(P)H FLIM [2], [4], [104], [180]. As a first step towards this goal, we made the assumption that the two cofactors possess finite and distinct fluorescence lifetimes when bound to enzymes inside tissue. We used computational modelling to show that the enzyme-bound lifetime of NAD(P)H fluorescence represents a concentration-weighted average of the specific enzyme populations present. By using HPLC assessment of the pyridine nucleotide contents of the NAD kinase overexpression and knock-down cell lines [87], we estimated the fluorescence lifetimes of intracellular bound NADPH and NADH to be 4.4(±0.2) ns and 1.5(±0.2) ns respectively [4]. As the fluorescence intensity of a population of molecules $\langle I_{\mathrm{total}}\rangle$ is dependent on both its concentration and its lifetime, FLIM allows fluorescence intensities to be converted into relative concentrations [184]. This allowed us to derive the following expressions for the concentrations of bound NADH and NADPH inside a tissue,

(1) $${\left[\mathrm{NADH}\right]}_{\mathrm{bound}}=k\left(\frac{4.4-\tau_{\mathrm{bound}}\left(\mathrm{ns}\right)}{2.9}\right)\frac{\langle I_{\mathrm{total}}\rangle }{\left(1-\alpha_{\mathrm{bound}}\right)\tau_{\mathrm{free}}+\alpha_{\mathrm{bound}}\tau_{\mathrm{bound}}}$$

(2) $${\left[\mathrm{NADPH}\right]}_{\mathrm{bound}}=k\left(\frac{\tau_{\mathrm{bound}}\left(\mathrm{ns}\right)-1.5}{2.9}\right)\frac{\langle I_{\mathrm{total}}\rangle }{\left(1-\alpha_{\mathrm{bound}}\right)\tau_{\mathrm{free}}+\alpha_{\mathrm{bound}}\tau_{\mathrm{bound}}}$$

These expressions rely on the arbitrary constant k, which is related to variables describing the experimental system such as the temporal profile of the illumination pulses, the efficiency of the detection apparatus and the absorption cross sections of NADH and NADPH [184]. Therefore in their present form, Eqs. (1), (2) allow only semi-quantitative assessment of the relative concentrations of NADH and NADPH. To make this technique fully quantitative to output concentrations in molar units, it is possible to measure k using a reference solution of NADH [133], [96].

Application of Eqs. (1), (2) allowed us to show that the redox ratio of the NADP pool is more oxidised in the nucleus than in the cytosol of HEK293 cells, while the redox state of the NAD pool is the same in the two compartments [4]. This is consistent with an absence of pentose phosphate pathway enzymes in the nucleus despite the presence of glycolytic enzymes [185]. We also used these formulae to show that EGCG treatment in the NAD kinase overexpressing cells affected only the bound NADPH and not bound NADH concentrations [4]. This reflected the specific inhibition of NADPH binding by EGCG [182], suggesting that this simple model may be a good first approximation to understanding the link between NAD(P)H biochemistry and intracellular fluorescence decay dynamics. Work is now continuing on developing more advanced models for assessing the abundances of the two pools using fluorescence lifetime measurements [186] in order to make this technique more widely accessible to the redox biology community.

## 4 Future directions in NAD(P)H autofluorescence

The coupling of ATP production to the reduction of oxygen to water in the mitochondria comes at the cost of oxidative stress. NAD and NADP lie at the heart of the balance between energy supply and ROS defence, and imbalance between the two leads to cellular dysfunction. Tools to study the behaviour of these cofactors in living tissues are therefore invaluable to our understanding of the role of mitochondrial redox state in health and disease.

Biochemical assays such as high-performance liquid chromatography (HPLC) offer direct measures of the redox state of NAD and NADP. However, imaging techniques based on the intrinsic fluorescence of these cofactors allow these measurements to be carried out in complex tissue preparations non-destructively, where differences are detectable between different cell types with subcellular resolution. Such methods may play an invaluable role in establishing the existence of novel metabolic coupling pathways between different cell types, such as the astrocyte-neuron lactate shuttle in the brain [187]. In addition to these advantages in basic biomedical research, further understanding the origins of variations in the fluorescence properties of NADH and NADPH in living tissues will also advance the field of autofluorescence diagnostics. While a range of clinical tools have been devised for imaging NAD(P)H fluorescence in situ [23], [24], [25], [26], [27], their practical application has been hindered by a lack of understanding of the metabolic meaning of the resulting data. Nevertheless, the crucial role of altered metabolism in pathogenesis and the centrality of NAD and NADP to these processes would suggest that an increased understanding of intracellular NAD(P)H fluorescence could contribute, alongside other laser-based techniques such as Raman spectroscopy [188], to the development of optical diagnostic devices.

Throughout this review, it has been clear that the pathways involved in determining the steady state NADH and NADPH levels represent a complex and interconnected network. The standard approach of applying pharmacological perturbations to a cell to isolate the contributions of NAD(P)H fluorescence from particular enzymatic subsystems can therefore be troublesome due to unexpected differential effects on neighbouring pathways. Under these circumstances, the understanding of NAD(P)H fluorescence changes may be aided by computational modelling [157], [189], [190]. Such approaches have already been applied successfully to intensity-based monitoring of redox state [191], but their adaptation to lifetime measurements is yet to be performed.

While our recent work has suggested that increased NADPH levels increase the mean fluorescence lifetime of enzyme-bound species, the reasons why this cofactor should display a longer fluorescence lifetime compared to that of NADH within the complex environment of the cell is not obvious. Differences in the fluorescence lifetime of NADH and NADPH when bound to their respective enzymes could be caused by variations in the level of conformational restriction of the cofactors in their corresponding binding sites [2]. However, discrimination between the two cofactors is realised at the adenine end of the molecule [179], away from the excited state localisation. Increased understanding of the photophysics of NADH and NADPH when bound to their respective enzymes will therefore be crucial in the ongoing advancement of the NAD(P)H FLIM field. Such knowledge will increase the level of metabolic information obtainable from these intrinsically fluorescent cofactors, and consequently enhance our understanding of mitochondrial redox state in health and disease.
